# Formation of equiaxed crystal structures in directionally solidified Al-Si alloys using Nb-based heterogeneous nuclei

**DOI:** 10.1038/srep39554

**Published:** 2016-12-23

**Authors:** Leandro Bolzoni, Mingxu Xia, Nadendla Hari Babu

**Affiliations:** 1 Waikato Centre for Advanced Materials, School of Engineering, The University of Waikato, Private Bag 3105, Hamilton 3240, New Zealand; 2 Shanghai Jiao Tong University, 800 Dong Chuan Road, Shanghai, 200240, China; 3 Brunel University London, Institute of Materials and Manufacturing, Kingston Lane, Uxbridge, Middlesex, UB8 3PH, United Kingdom

## Abstract

The design of chemical compositions containing potent nuclei for the enhancement of heterogeneous nucleation in aluminium, especially cast alloys such as Al-Si alloys, is a matter of importance in order to achieve homogeneous properties in castings with complex geometries. We identified that Al_3_Nb/NbB_2_ compounds are effective heterogeneous nuclei and are successfully produced in the form of Al-2Nb-xB (x = 0.5, 1 and 2) master alloys. Our study shows that the inoculation of Al-10Si braze alloy with these compounds effectively promotes the heterogeneous nucleation of primary α-Al crystals and reduces the undercooling needed for solidification to take place. Moreover, we present evidences that these Nb-based compounds prevent the growth of columnar crystals and permit to obtain, for the first time, fine and equiaxed crystals in directionally solidified Al-10Si braze alloy. As a consequence of the potent heterogeneous particles, the size of the α-Al crystals was found to be less dependent on the processing conditions, especially the thermal gradient. Finally, we also demonstrate that the enhanced nucleation leads to the refinement of secondary phases such as eutectic silicon and primary silicon particles.

Al-Si alloys are important structural materials commonly processed by means of casting methods due to their good fluidity^1^. Al-Si alloys are commonly used in a great variety of applications like automotive components (engine parts and wheels), marine fittings, architectural panels as well as medical and dental equipment^2^,^3^. The shape of these cast products such as engine blocks is generally rather complex and thus they are composed of sections with different wall thicknesses. Under standard solidification conditions, the cast part exhibits a non-uniform grain structure. Specifically, thicker sections solidify under slower cooling rates (i.e. smaller thermal gradient) and thus will be characterised by coarser structures in comparison to thinner sections^4^. As the grain structure is related to the mechanical behaviour of the material, the cast part has an anisotropic response when withstanding external loads such as mechanical or thermal stresses causing its premature failure. The achievement of fine equiaxed grains is desirable in metals casting, as benefits in terms of increased soundness of the castings (i.e. lower rejection rate) due to the better feeding of the molten metal in the mushy zone resulting in more homogeneously distributed porosity and reduced hot tearing susceptibility, higher mechanical performances, and easier and more homogeneous subsequent mechanical working are obtained. A structure characterised by a fine equiaxed crystal structure independently of the cooling conditions (i.e. thickness of the walls of the part) would also open up the possibility to improve and optimise the design of the castings.

The formation of a non-uniform grain structure is particularly prominent in directionally solidified parts such as DC-casting (i.e. direct chill) billets, whose diameter can be in the range of meters. In directionally solidified materials the solidification starts from the outer surface (called chilled zone) and proceeds toward the centre of the casting. Because of the intrinsic nature of the solidification process, the casting is composed of columnar grains in its outer part changing to equiaxed crystals moving towards its centre. This difference in crystal structure along the cross section of the casting negatively affects the response of the material during subsequent downstream deformation processes via rolling, extrusion, etc. For example, the Al-10Si braze alloy is normally obtained as DC-casting billet and then plastically deformed to manufacture cars’ radiators. Consequently, the achievement of (fine) fully equiaxed grain structures is also paramount in directional solidified components in order to guarantee isotropic deformation behaviour and prevent the formation of defects and cracks^5^. Specifically, reduction of billets scalping and increased yield of expensive post-processed material are the advantages of having fine and uniform crystal structures.

As per the classical nucleation theory, more uniform grain structures can be achieved if potent nuclei are present to favour the heterogeneous nucleation of the crystals of the primary phase^6^. Furthermore, the promotion of nucleation over growth (i.e. the two stages of solidification) results in the attainment of a greater number of fine crystals, independently of the local solidification conditions. Accordingly, improved properties and performances (regardless whether they are physical, mechanical or technological) as well a more isotropic behaviour of the material are also obtained^3^,^7^,^8^. The intentional addition of heterogeneous nuclei to promote nucleation is known as inoculation.

The energy barrier (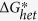) for nucleation of a molten metal inoculated with heterogeneous nuclei is expressed in thermodynamic terms as (1):


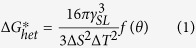


where *γ*_*SL*_ is the solid-liquid interface energy, Δ*S* is the variation in entropy, Δ*T* is the variation in temperature and *f(θ)* is a function of wetting angle *(θ)*. Δ*T* is also known as the undercooling needed for solidification to take place. From (1), 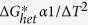 where Δ*T* is proportional to the square of the lattice mismatch (**δ**) between the heterogeneous nuclei and the nucleating phase (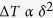), as reported by Turnbull and Vonnegut^9^. Hence, heterogeneous nuclei with high potency to promote nucleation must have good lattice and atom position matching with the nucleating phase (i.e. low **δ**). Other two aspects should be taken into account to design effective heterogeneous nuclei for the solidification of materials^10^: the nuclei must have a sufficiently high melting point as not to dissolve in the molten material and the nuclei must be chemically stable so its lattice structure will not change once the nuclei are in contact with the chemical elements present in the molten material. A typical example of the interaction of intentionally added heterogeneous nuclei with the alloying elements of the molten material does happen when Al-Ti-B or Al-Ti-C master alloys are used to inoculate Al-Si alloys. These Ti-based master alloys, which are very efficient in promoting the nucleation in wrought Al alloys, are not effective in Al-Si alloys because the Ti-based nuclei react with the silicon of the Al-Si alloy to form titanium silicides (i.e. TiSi, Ti_5_Si_3_ and TiSi_2_)^11^,^12^,^13^,^14^,^15^. These titanium silicides have high **δ** with the Al lattice and therefore nucleation cannot be promoted. Moreover, it has been scientifically proven via thermodynamic studies that the excess Ti, compared to what is needed to form nucleating TiB_2_ particles, increases the growth rate of Al-dendrites so that growth wins over nucleation^16^,^17^,^18^,^19^. We discovered that Nb-based compounds (Al_3_Nb/NbB_2_) are not characterised by this phenomenon, at least at the processing temperature conventionally used in aluminium foundries (~700–800 °C). This is because the formation of niobium silicides occurs at much higher temperatures in comparison to titanium silicides. This means that Al_3_Nb/NbB_2_ are much more thermodynamically and chemically stable^20^,^21^ in comparison to Al_3_Ti/TiB_2_. Previously, we demonstrated that Al_3_Nb/NbB_2_ can promote the nucleation of Al crystals in Al-Si alloys processed by die casting^22^,^23^; however, the formation of uniform grain structures in directionally solidified Al-Si casting has not been achieved before. Al-10Si braze alloys are typically processed using DC casting process in which the melt undergoes directional solidification imposed by cooling by water spray. Large columnar grains formation is not ideal due to the non-uniform chemical composition and the need for scalping large portion of billet. In this work, we designed and successfully achieved enhanced heterogeneous nucleation of Al-10Si alloys via inoculation by means of Nb-based compounds using Al-2Nb-xB (x = 0.5, 1 and 2) master alloys. Most importantly, a comprehensive understanding of the effects and phenomena taking place during the inoculation of Al-Si alloys with Nb-based compounds is presented. In particular, the directional solidification of an Al-10Si braze alloy using an equipment which simulates the industrial scale DC-casting method was considered. The presence of Al_3_Nb/NbB_2_ heterogeneous nuclei resulted in very fine uniform grain structure across the casting.

## Characterisation of the Al-2Nb-xB (x  =  0.5, 1 and 2) Master Alloys

**Figure 1:**
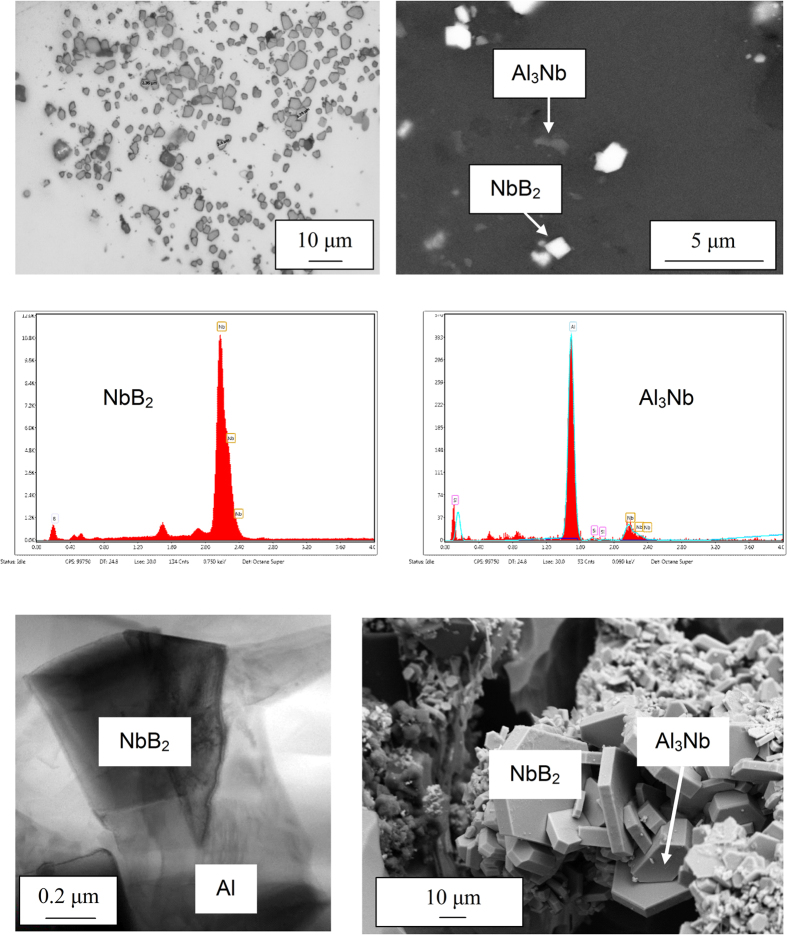
Representative results of the microstructural characterisation performed on the Al-2Nb-xB master alloys: (**a**,**b**) optical and secondary electron micrographs showing the distribution of the Nb-based compounds embedded into the Al matrix, (**c**,**d**) semi-quantitative EDS analysis of the chemistry and stoichiometry of the Nb-based compounds, and (**e**,**f**) TEM and SEM micrographs of deep etched Al-2Nb-xB master alloys showing the tetragonal and hexagonal crystallographic structure of Al_3_Nb and NbB_2_ crystals, respectively.

Al, Nb and an Al-5B master alloy were used to produce the Al-2Nb-xB master alloys (x = 0.5, 1 and 2) containing the Nb-based compounds (Al_3_Nb/NbB_2_). These compounds should remain as stable crystals dispersed into the Al matrix once the Al-2Nb-xB master alloy is cast. Al_3_Nb/NbB_2_ crystals act as heterogeneous nuclei once the Al-2Nb-xB master alloy is added to the molten Al-Si cast alloys prior to their solidification. The actual formation and presence of these Nb-based compounds was confirmed via SEM-EDS and TEM analyses of the Al-2Nb-xB master alloys (Fig. 1).

A fairly homogeneous distribution of sub-micrometric to micrometers Nb-based compounds embedded into the Al matrix was found. Moreover, instead of coarse single crystals, agglomerations of Al_3_Nb/NbB_2_ crystals were formed as a consequence of the production route used to manufacture the Al-2Nb-xB master alloys. These agglomerates are present in the concentrated master alloy and when the master alloy is added to Al-10Si melt, inoculants disperse in to the alloy melt. For higher concentrations of x (1 and 2), in addition to Al_3_Nb/NbB_2_ compounds, unreacted AlB_12_ blocky particles were present in the master alloy. TEM and SEM microstructural analysis of the deep etched Al-2Nb-xB master alloys was conducted to study the crystallographic structure: tetragonal for Al_3_Nb (DO_22_)^24^,^25^ and hexagonal for NbB_2_ (P_6/mmm_)^26^. Lattice parameters of these structures are: a = 3.8485 Å and c = 8.615 Å for Al_3_Nb and a = 3.102 Å and c = 3.285 Å for NbB_2_, respectively^27^. The semi-quantitative EDS chemical analysis further proved the chemistry and stoichiometry of the Nb-based compounds.

## Solidification Experiments

**Figure 2:**
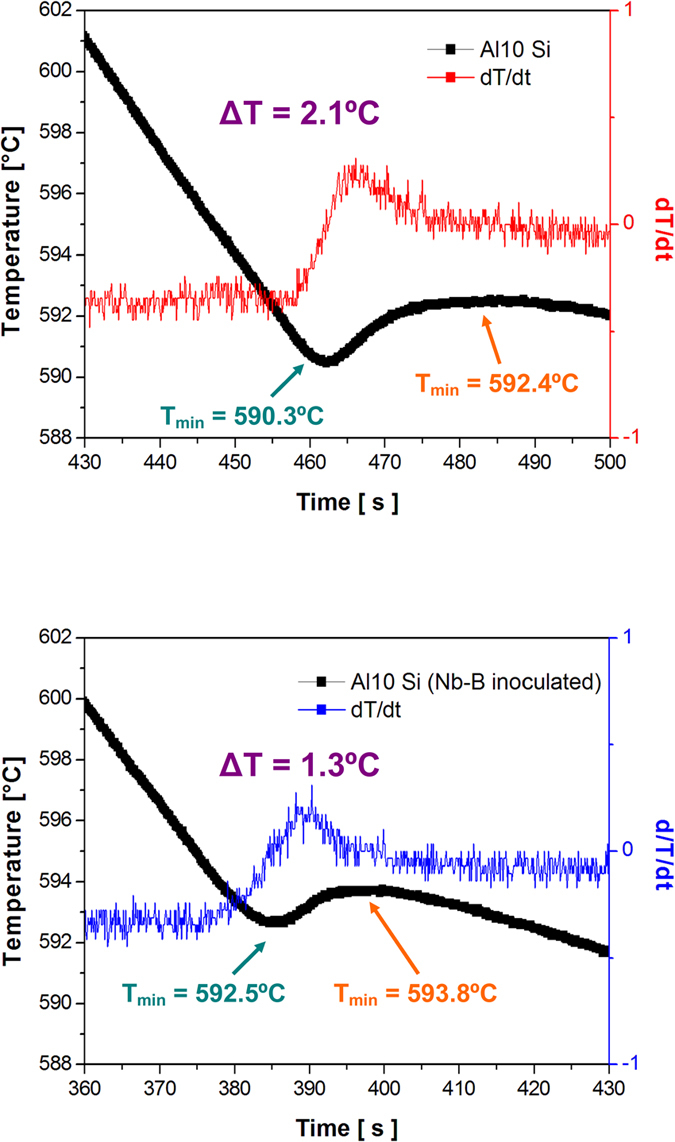
Solidification curves of the Al-10Si alloy without (**a**) and with (**b**) inoculation by means of Nb-based heterogeneous nuclei as to reduce the undercooling (*ΔT*) needed to commence the nucleation of primary α-Al crystals.

Thermal analysis of the solidification of the Al-10Si alloy was performed in order to quantify the effect of the inoculation by means of Nb-based heterogeneous nuclei. The total undercooling (Δ*T*) needed to have a sufficiently large stable cluster of atoms to nucleate primary α-Al in the reference material is 2.1 °C (Fig. 2). The inoculation of the Al-10Si alloy via the addition of the Al-2Nb-xB master alloys lowers Δ*T* down to 1.3 °C, therefore reducing 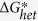 by approximately 40% and enhancing the formation of primary α-Al crystals.

**Figure 3:**
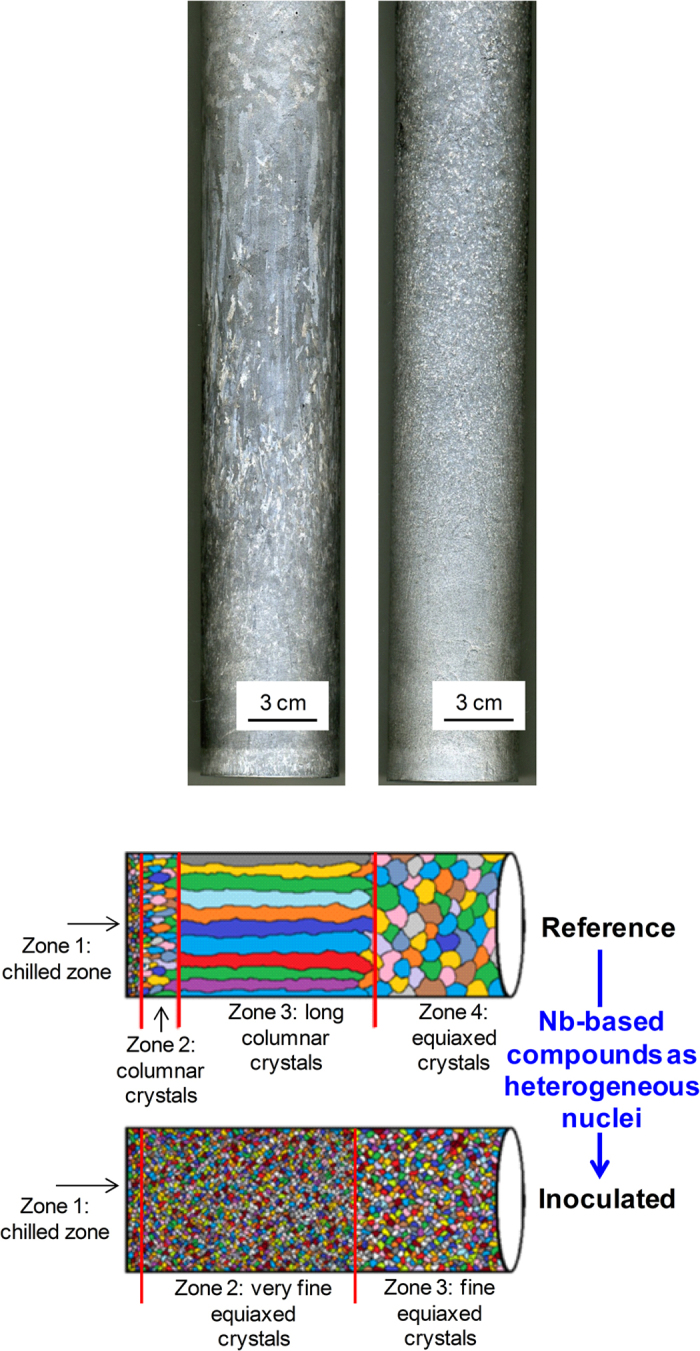
Macroetched surface of directionally solidified Al-10Si billets produced using the DC-casting simulator: (**a**) without inoculation, (**b**) with inoculation by means of Nb-based heterogeneous nuclei, and (**c**) schematic of the different grain structures.

The inoculation of the Al-10Si alloy prior to directional solidification led to a significantly different crystallographic structure compared to the reference alloy (i.e. different solidification zones and relative variation of the nature and size of the crystals formed) as presented in Fig. 3.

**Figure 4:**
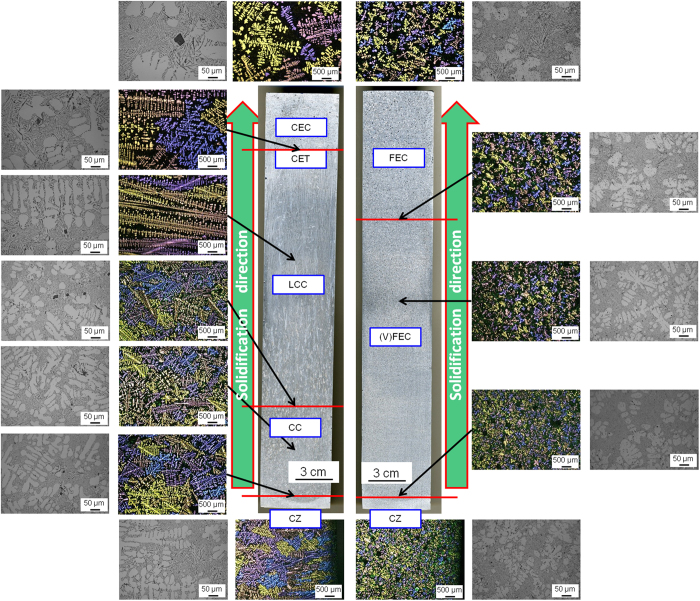
Results of the microstructural analysis of the directionally solidified Al-10Si alloy showing the different crystallographic structures: (**a**) without inoculation and (**b**) with inoculation by means of Nb-based heterogeneous nuclei. The introduction of Nb-based compounds significantly enhances nucleation promoting the formation of equiaxed crystals and preventing the formation of columnar grains; this results in a much more isotropic crystallographic structure. As a consequence, better deformation behaviour in subsequent downstream processes and improved performances (technological and mechanical) are predicted. (Legend - CZ: chilled zone, CC: columnar crystals, LCC: long columnar crystals, CET: columnar-to-equiaxed transition zone, CEC: coarse equiaxed crystals and (V) FEC: (very) fine equiaxed crystals).

The directional solidified Al-10Si reference alloy is characterised by a non-uniform grain structure composed of 4 zones. After inoculation by means of Nb-based heterogeneous nuclei the formation of columnar grains is prevented, hence the columnar to equiaxed transition (CET), and the microstructure is composed of equiaxed crystals whose size slightly progressively coarsen along the direction of solidification. The microstructural characterization performed on the polished and etched cross sections of the billets confirmed the different crystal structures of the Al-10Si alloys (Fig. 4).

**Figure 5:**
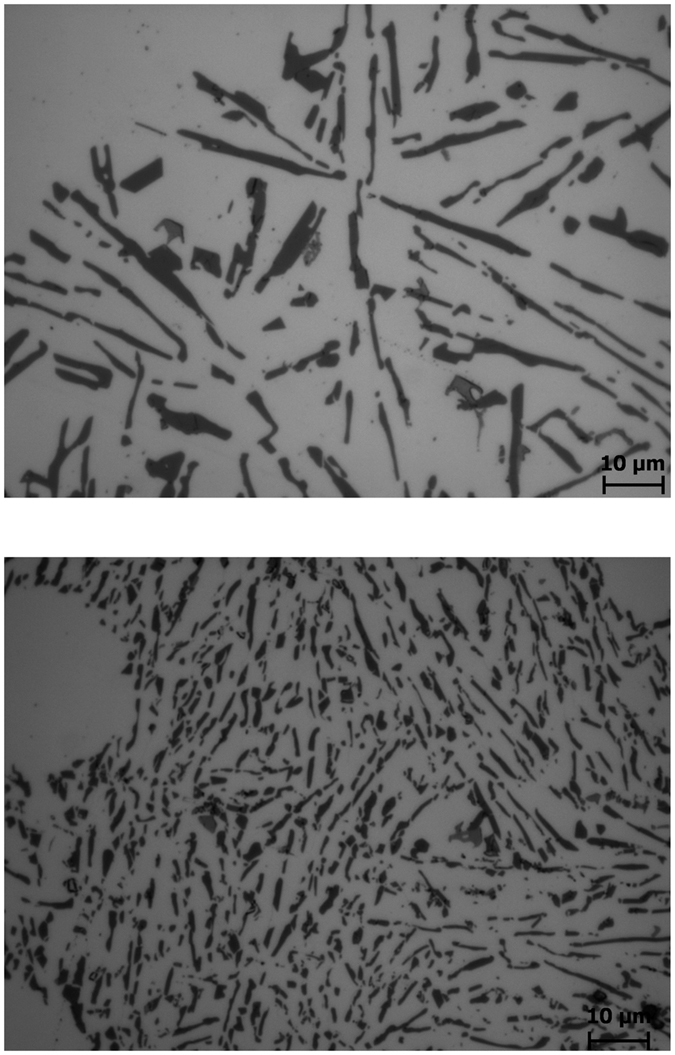
Details of the secondary eutectic phase of the directionally solidified Al-10Si alloys: (**a**) without inoculation and (**b**) with inoculation by means of Nb-based heterogeneous nuclei. The micrographs refer to the CEC and FEC zones described in Fig. 4, respectively.

Apart from α-Al crystals, the microstructure of the directionally solidified Al-10Si alloy is composed of needle-like eutectic Si, which are actually flakes in 3D, and primary Si particles. In the case of the reference alloy, a non-uniform distribution of the eutectic phase (50–200 μm) is formed in between the dendritic arms of the growing columnar and equiaxed crystals (Fig. 5a). Moreover, faceted primary Si particles (20–50 μm) are also present as the partitioning of Si becomes more important with the progression of the solidification. The number and size of these primary Si particles is thereby greater in the CEC zone of the reference alloy than in the CC and LCC zones (Fig. 4a). The Al-10Si alloy inoculated with Nb-based compounds shows a more uniform distribution of finer secondary eutectic phase (10–40 μm) as a consequence of the finer α-Al crystals (Fig. 5b). Furthermore, the size and number of the primary Si particles (<10 μm) is significantly lower (Fig. 4b).

## Discussion

Nb-based compounds (Al_3_Nb/NbB_2_) are characterised by the same crystallographic structures of the Ti-based compounds (Al_3_Ti/TiB_2_) used to refine wrought Al alloys. Specifically, Al_3_Ti and Al_3_Nb crystallises into the tetragonal lattice whereas TiB_2_ and NbB_2_ have hexagonal lattice (Fig. 1). Apart from being isomorphous, Ti- and Nb-based compounds have also similar lattice parameters and therefore comparable lattice mismatch *(*δ*)* with the face-centred-cubic lattice of Al. The low **δ** is one of the critical factors to promote heterogeneous nucleation. As a consequence of the low **δ**, a reduction of the undercooling *ΔT* is expected^8^,^28^,^29^. The solidification experiments done on the Al-10Si alloy confirmed that the addition of engineered nuclei, actually, reduces *ΔT* (Fig. 2) due to the low ***δ*** between Al_3_Nb/NbB_2_ and primary α-Al as 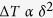. In particular, as per Bramfitt’s model for heterogeneous nucleation^30^, the nucleation of primary α-Al crystals is likely to happen on: Al_3_Nb crystals, from an Al_3_Nb layer formed on top of NbB_2_ compounds (such as in the case of Ti-based nuclei^31^) as well as along low index planes of NbB_2_ crystlas.

During directional solidification, the Al-10Si reference alloy undergoes different changes in crystal morphology (Fig. 3a) forming a non-uniform and complex crystal structure composed of four different zones (Fig. 4a). At the bottom of the billet, the alloy starts to solidify in contact with the Cu-plate and immediately afterwards, once the Cu-plate is removed, comes into direct contact with the water jet. Consequently, due to the high thermal gradient (i.e. cooling rate) this zone is characterised by extremely fine randomly oriented dendritic crystals (Zone 1: CZ). Starting from these non-faceted fine crystals, grains with specific orientation growing along the direction of the extraction of the latent heat prevail over the others. Thus, the microstructure of the Al-10Si alloy starts to be composed of a greater number of columnar crystals of relatively small size (Zone 2: CC). Afterwards, relatively few long columnar crystals survive and constitute the microstructure of the material (Zone 3: LCC). The formation of columnar crystals is due to different factors such as constrained growth imposed by the columnar crystals, solute partitioning, thermal gradient, latent heat extraction as well as the competition between the different growing crystals. At this stage, the predominant solidification mechanism is still columnar dendritic as it was in Zone 2 and Zone 3. With the progression of directional solidification, the crystal structure of the material transforms to equiaxed (Zone 4: CEC), although the crystal size is relatively coarse. The CET is governed by the reduction of the thermal gradient combined with the lower growth rate and the formation of new stable nuclei due to solute enrichment. The formation of these randomly oriented crystals indicates that in the final stage the solidification mechanism is purely equiaxed dendritic. The behaviour described is consistent with the current understanding of microstructural evolution in the DC-casting process^5^.

In the case of the Al-10Si alloy inoculated by means of Nb-based heterogeneous nuclei, the chilled zone composed of randomly oriented crystals is still present (Zone 1: CZ). Once again, the formation of this zone is governed by equiaxed solidification by heterogeneous nucleation due to the walls of the die and the fast heat exchange (i.e. extraction by conduction) due to the water jet. Conversely to the reference material, after CZ the microstructure of the inoculated Al-10Si alloy is characterised by the presence of very fine equiaxed crystals (Zone 2: VFEC) and the formation of columnar crystals is prevented thanks to the presence of the engineered heterogeneous nuclei (Fig. 4b). Finally, due to some solute enrichment (i.e. silicon) in combination with the remelting of nuclei and dissolution, dendrite coarsening occurs in the final section of the billet to solidify (Zone 3: FEC). Nevertheless, these crystals are much smaller than those of the reference material. Therefore, it is evident that the introduction of the heterogeneous nuclei prevents the columnar dendritic growth and promotes the nucleation of fine equiaxed α-Al dendritic crystals.

The formation of the constrained-growth columnar crystals along the direction of heat extraction during directional solidification is commonly described by means of the classical solidification theory^32^:


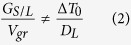


where *G*_*S/L*_ is the thermal gradient at the solid-liquid interface, *V*_*gr*_ is the growth velocity, *ΔT*_*0*_ is the (constitutional) undercooling and *D*_*L*_ is the diffusion coefficient of the liquid metal. Depending on whether the left hand side term is higher/equal or lower than the right hand side term, planar or cellular/dendritic growth will occur, respectively. The columnar dendritic structure found in the reference material is due to the high *G*_*S/L*_ present in the directional solidification of the billet. As the columnar growth progresses, *G*_*S/L*_ and *V*_*gr*_ decreases. *ΔT*_*0*_ promotes the formation of new crystals ahead of the solidification front which contributes to latent heat release and solute enrichment, changing the growth mechanism to equiaxed dendritic. The formation of a fully columnar or fully equiaxed crystallographic structure depends on *G*_*S/L*_ and the total number of nuclei per unit volume (*N*_*0*_):


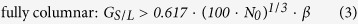



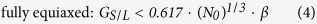


where *β* is a function of the critical *ΔT* for nucleation and of the local *ΔT* at the columnar front. From (3) and (4), equiaxed growth will occur when the volume fraction of equiaxed grains is greater than 0.49. The number of nuclei per unit volume *N*_*0*_ can be estimated from the grain size^33^. In the case of the LCC zone (Fig. 4) of the reference Al-10Si alloy, *N*_*0*_ is ~1 grain/mm^3^ whereas after inoculation, at the same position in the directionally solidified alloy, *N*_*0*_ is ~24 grains/mm^3^. From this, it is inferred that the prevention of the formation of constrained-growth columnar grains in the inoculated material is a direct consequence of the presence of potent heterogeneous nuclei ahead of the growth front. The formation of these heterogeneous nucleated equiaxed crystals also leads to a greater homogeneous distribution of the solute (i.e. silicon) in the melting pool ahead of the solidification front which lowers *ΔT*. Concerning the eutectic phase that forms upon directional solidification of the Al-10Si, in the case of the reference material, the size and distribution of this secondary phase becomes coarser and more inhomogeneous along the cross section of the billet. The size and distribution of the eutectic phase is a direct consequence of the nucleation and growth of the primary α-Al dendritic crystals as solute remains confined to regions between the growing crystals independently whether they have a columnar or equiaxed morphology^4^,^34^. The coarsening of the eutectic phase is also influenced by the enrichment in solute which is rejected and pushed by the solidification front. This means that the local content of Si increases leading to the formation of coarse eutectic needles and/or flakes (Fig. 5a). Furthermore, in the case of the reference alloy, the local Si concentration in the eutectic pools between the dendritic arms of the growing α-Al crystals exceeds the eutectic composition (i.e. 12.6 wt.% as per the Al-Si binary phase diagram^35^). As a result, primary Si crystals (faceted, black and blocky crystals visible in the micrographs shown in Fig. 4a) are found in the Al-10Si alloy despite of the fact that it is a hypo-eutectic alloy. It is worth mentioning that both the size and number of these primary Si crystals becomes larger moving from CZ towards LCC/CEC. In particular, the larger Si crystals are found in the final section of the billet to solidify (i.e. CEC zone in Fig. 4) because it is the zone which is richest in rejected Si solute. The inoculation of the Al-10Si alloy by means of Nb-based heterogeneous nuclei results in the formation of finer eutectic Si as shown in Fig. 5. Moreover, their distribution is more homogeneous along the whole cross section of the directionally solidified inoculated material. Another aspect to be highlighted is the reduction in size and the lower number of primary Si crystals that form after inoculation. The Nb-based compounds that act as a heterogeneous nuclei for the α-Al crystals should not promote the nucleation of the primary Si crystals because the lattice structure (i.e. parameters and **δ**) are not favourable. Hence, the refinement of the eutectic and primary Si particles is attributed to the more homogeneous distribution of the Si solute at the equiaxed solidification front as well as to the growth restriction factor imposed by the formation of a much greater number of primary α-Al crystals leaving less space for the eutectic Si to grow. These aspects are also responsible for the formation of a lower amount of the primary Si crystals whose size is smaller than in the case of the reference Al-10Si alloy.

The current study demonstrates that the inoculation of cast Al-Si alloys via the introduction of Nb-based compounds (Al_3_Nb/NbB_2_) using Al-2Nb-xB master alloys enhances the heterogeneous nucleation of the material. Consequently, the directionally solidified grain structure is not greatly influenced by the processing conditions (e.g. cooling rate, heat extraction, thermal gradient, growth velocity, etc.). The more isotropic behaviour of the inoculated material allows fabrication of geometrically complex products (i.e. with differences in walls thicknesses) with homogeneous properties. Furthermore, optimised and lighter structures could be produced due to the enhanced behaviour of the inoculated alloys. These aspects are particularly important and desirable for the automotive industry where the stringent regulations for decreasing the greenhouses gases emission is pushing this sector toward the use of lightweight structural components. Cast Al-Si alloys with non-optimised crystals structures are already extensively used for the production of engine parts such as engine blocks, pistons and wheels. Further weight savings will be achieved by obtaining fine and homogeneous grain structures via inoculation by means of Nb-based heterogeneous nuclei.

From the study it can be concluded that an effective approach was developed to enhance heterogeneous nucleation in Al-Si cast alloys as demonstrated in directionally solidified materials. The Nb-based compounds introduced via the addition of the Al-2Nb-xB master alloys effectively act as heterogeneous nuclei due to their low lattice mismatch with the Al lattice and they are thermodynamically and chemically stable inside the melt. The inoculation of Al-Si cast alloys by means of Al_3_Nb/NbB_2_ prevents the formation of (long) columnar crystals thus permitting to achieve a highly homogeneous crystal structure, both in terms of primary α-Al crystals and secondary phases (eutectic and primary Si crystals). It is foreseen that this translates into a more isotropic material with improved behaviour making it suitable to fabricate high performance braze alloys and also more optimised structural components using various Al-Si alloys.

## Methods

### Production and characterisation of Al-2Nb-xB (x = 0.5, 1 and 2) master alloys

**Figure 6:**
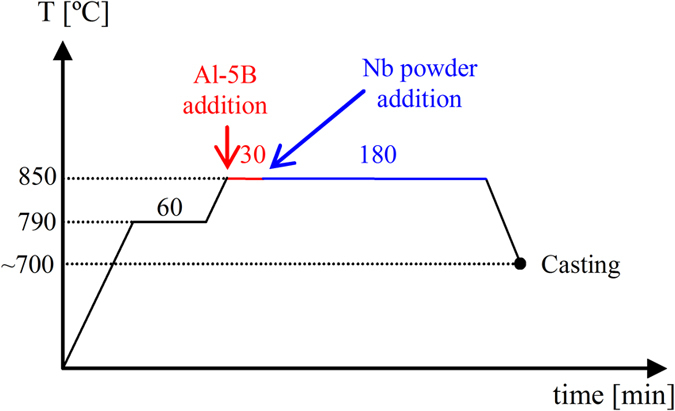
Thermal cycle (temperature-time) used for the fabrication of Al-2Nb-xB (x = 0.5, 1 and 2) master alloys.

The Al-2Nb-xB (x = 0.5, 1, and 2) master alloys were produced by melting commercially pure Al to which an Al-5B master alloy and Nb powder were added. For the dissolution of Nb and the formation of Nb-based compounds (Al_3_Nb/NbB_2_), the master alloy was kept at 850 °C during 3 hours with intermediate manual stirring every 15 minutes (Fig. 6) as it proved to be efficient to produced stable Nb-based compounds^36^.

Microstructural analysis of the master alloy was done on a JEOL 2200F-TEM and on a Zeiss Supra 35VP FEG microscope in order to confirm the formation and presence of the Nb-based compounds. EDS was performed to obtain semi-quantitative chemical composition data.

### Solidification experiments

Solidification experiments were done using a commercial Al-10Si braze alloy (Si = 9.9 wt.%, Fe = 0.09 wt.% and Al = balance). Initially, the Al-10Si alloy melt without and with the addition of the Al-2Nb-xB master alloys was solidified under slow cooling conditions (insulated crucible ~0.35 °C/s) to verify and quantify the effect of the presence of the engineered heterogeneous nuclei on the undercooling needed for nucleation. It is worth mentioning that in these experiments, *T*_*min*_ and *T*_*g*_ are the minimum and maximum temperature of the undercooling peak (Fig. 2). *T*_*min*_ is the point where the latent heat of fusion is given off and *T*_*g*_ is the point at which the steady growth state is reached. The difference between these two temperatures is defined as the undercooling:


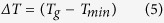


**Figure 7:**
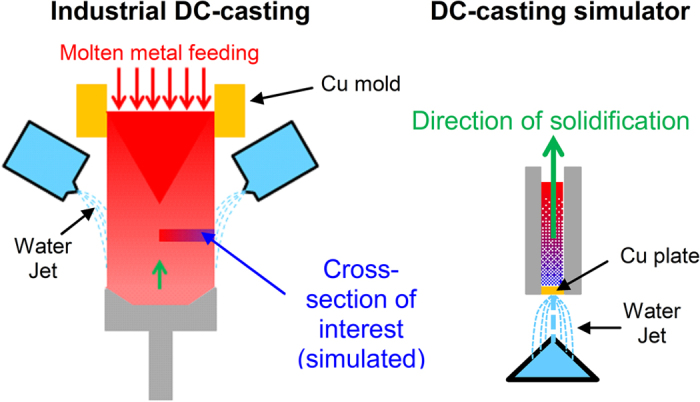
Schematic of the industrial directional solidification DC-casting process (**a**) where the cross section simulated is highlighted inside the industrial ingot/billet and (**b**) schematic of the directional solidification, which simulates the casting conditions in the highlighted cross section in industrial DC-casting billets.

Directional solidification experiments were done using a DC-casting simulator and for that the Al-10Si alloy was melted in a clay-bonded graphite crucible at 790 °C for 1 hour. In conventional industrial DC-casting of ingots and billets the directional solidification of the molten alloy takes place from the outer diameter towards the inner core of the billet aided by cooling from a water jet. In order to simulate this cooling system, the molten Al-10Si alloy was poured inside an insulated metallic cylinder and cooled from the bottom by means of a water jet. This can be seen in Fig. 7, which shows a schematic of both the industrial DC-casting process and the DC-casting simulator. In the latter case the cooling of the molten metal takes place along the length of the billet. The resulting microstructure is, therefore, the simulation of the cross section of very large billets or ingots.

In the case of the reference material, the Al-10Si alloy was poured into the DC-casting simulator and cooled/chilled from 740 ± 3 °C. The same experimental procedure was used for the Al-10Si alloy inoculated by means of the Al-2Nb-xB master alloys (addition level equivalent to 0.1 wt.% of niobium). The master alloy was left in contact with the melt for 15 minutes prior pouring of the alloy in the DC-casting simulator. For all Al-2Nb-xB (for x = 0.5, 1, and 2) master alloys addition, the billets were composed of equiaxed crystals and the data and discussion presented here is based on x = 2 as representative for all the Al-2Nb-xB master alloys.

On the one side, analysis of the macrostructure of the cast billets was done by chemically etching the surface of the billets using Tuckers solution (15 ml HF + 15 ml HNO_3_ + 45ml HCl + 25ml H_2_O). On the other side, microstructural analysis was performed on polished and etched samples using a Zeiss Axioscope A1 microscope. Light polarised micrographs were taken on samples anodised using a tetrafluoroboric acid (HBF_4_) solution and passing a current of approximately 10 V/1 A.

## Additional Information

**How to cite this article**: Bolzoni, L. *et al*. Formation of equiaxed crystal structures in directionally solidified Al-Si alloys using Nb-based heterogeneous nuclei. *Sci. Rep.* **6**, 39554; doi: 10.1038/srep39554 (2016).

**Publisher's note:** Springer Nature remains neutral with regard to jurisdictional claims in published maps and institutional affiliations.
